# Social-ecological goals and outcomes of public engagement for recovery of endangered and threatened rockfishes (*Sebastes* spp.)

**DOI:** 10.1371/journal.pone.0331686

**Published:** 2025-09-09

**Authors:** Anne H. Beaudreau, Dayv Lowry, Jennifer Blaine, James Selleck, Robert Pacunski, Kathryn Meyer

**Affiliations:** 1 School of Marine and Environmental Affairs, University of Washington, Seattle, Washington, United States of America; 2 Protected Resources Division, West Coast Region, National Marine Fisheries Service, National Oceanic and Atmospheric Administration, Lacey, Washington, United States of America; 3 Washington Department of Fish and Wildlife, Olympia, Washington, United States of America; 4 Natural Resources Consultants, Inc., under contract to Protected Resources Division, West Coast Region, National Marine Fisheries Service, National Oceanic and Atmospheric Administration, Seattle, Washington, United States of America; MARE – Marine and Environmental Sciences Centre, PORTUGAL

## Abstract

Natural resource management agencies commonly conduct outreach and engagement with the public, with the goals of raising awareness, educating constituents, encouraging compliance with rules, and supporting future participation in management processes. In Washington, USA, significant effort was invested over more than a decade to inform and engage recreational anglers and divers, and the broader public, in recovery efforts related to rockfish species (*Sebastes* spp.) listed for protection under the Endangered Species Act (ESA). We developed a novel evaluative framework using mixed methods to assess outreach and engagement efforts related to ESA-listed rockfishes from 2010 to 2022. We triangulated qualitative and quantitative data from interviews and surveys to: summarize the scope, content, goals, and intended audiences for rockfish-related outreach and engagement; examine practitioner perspectives regarding the effectiveness and challenges of rockfish-related outreach and engagement; assess anglers’ current understanding about rockfish management status and fishing regulations, drawing comparisons to the time of the ESA listings where possible; and synthesize strengths and weaknesses of past outreach and engagement to guide future efforts. We found that a portfolio of outreach and engagement approaches will likely be most effective for reaching a diverse audience and meeting multiple social goals. Additionally, community-engaged collaborations allow for a deeper discourse that builds trust, knowledge, and shared stewardship. Finally, strategic partnerships and capacity-building will continue to be important for meeting outreach and engagement goals. These results will help guide future efforts that are tailored to best meet the evolving needs of rockfish recovery.

## Introduction

Public participation plays an important role in environmental decision-making, spanning from formal public processes to voluntary partnerships between government agencies and engaged community members [1]. In natural resource management, a variety of legal mandates and institutional goals have translated into a broad typology of participation [2,3]. These commonly include multiple forms of unidirectional communication from government bodies to the public and vice versa, such as education and outreach efforts by agencies to inform the public about regulations, or written and oral comments provided by community members to decision-makers during public hearings [4]. Two-way engagement, such as public participation on advisory bodies and deliberative participatory processes, also commonly plays a role in decision-making settings [4,5]. Public participatory processes have had variable outcomes, with some downsides including increased costs and time for decision-making and challenges in ensuring participation of diverse groups [6,7]. More often, participation has been demonstrated to increase trust, credibility, and acceptability of management decisions, and to improve the quality of decision-making by engaging local knowledge and expertise in problem-solving [3,6,8].

Multiple modes of outreach and engagement by management agencies may be necessary for meeting a range of social goals for public participation [9]; however, the effectiveness of these efforts can be difficult to assess (e.g., [10]), or may not be assessed at all [4,11]. This creates a challenge for prioritization and funding of future outreach and engagement efforts. Quantitative and qualitative frameworks for assessing the effectiveness of outreach and engagement offer differing strengths and weaknesses. Surveys aimed at evaluating stakeholder knowledge, attitudes, and behavior before and after involvement can provide information on causal effects of outreach (e.g., [12,13]) but may be affected by non-response bias [14] and can lack contextual information needed to understand why the effectiveness of particular approaches varied. Qualitative methods, such as focus groups or interviews with agency staff and resource users, can provide this valuable contextual information [15], but usually have a more limited number of study participants and may overrepresent perspectives of highly engaged individuals who choose to participate. Combining quantitative and qualitative approaches to evaluate effectiveness of outreach and community engagement in natural resource management settings can provide a more holistic framework to guide future efforts.

In this study, we combined interviews with outreach practitioners and surveys of anglers to evaluate the effectiveness of a decade of outreach and engagement related to recovery of endangered and threatened rockfishes (*Sebastes* spp.). Rockfish are a highly diverse group of long-lived fishes found along the west coast of North America that declined throughout the 20^th^ century due to overharvest and a mix of other anthropogenic and environmental pressures [16–19]. Among the key challenges in rockfish conservation and management are: 1) slow growth and long generation time of rockfish, leading to long recovery horizons; 2) susceptibility of many rockfish species to localized depletion due to small home ranges and high site fidelity; 3) data limitations arising from challenges with *in situ* monitoring and species identification; and 4) the sensitivity of rockfish to injury resulting from rapid ascent from deep water (i.e., barotrauma) [16]. Thus, education efforts aimed at improving rockfish identification by fishers and promoting handling practices that minimize post-release mortality have long been a focus of management agencies along the west coast of North America [20–25].

In the Salish Sea, a large semi-enclosed estuary in the Pacific Northwest region of North America, three species of rockfish were listed for protection under the ESA in 2010 (75 FR 22276). Bocaccio (*S. paucispinis*) was listed as endangered, and yelloweye rockfish (*S. ruberrimus*) and canary rockfish (*S. pinniger*) were listed as threatened. Canary rockfish was delisted in 2017 (82 FR 7711−7731) as a result of new genetic information indicating that the Salish Sea population is not distinct from the outer coast [26,27]. The last commercial rockfish catch in Puget Sound, a basin within the Salish Sea, was recorded in 1993 [18]. Following the ESA listing, a moratorium was placed on retention of recreationally caught rockfish in most management areas (WAC 220-314-020), and bottomfish fishing for most non-rockfish species was restricted to depths shallower than 120 feet (36.6 m) (NMFS 2017, WAC 220-314-010). In 2017, a regulation was adopted that required a descending device to be onboard and rigged for immediate use on recreational vessels targeting bottomfish and halibut (WAC 220-310-110). A federal Rockfish Recovery Plan published in 2017 identified five recovery actions [22]. These included conducting education, outreach, and public engagement focused on: a) improving rockfish identification and documentation of bycatch by fishers; b) encouraging avoidance of rockfish and increased use of descending devices; c) improving knowledge of rockfish life history and ecology; d) improving public understanding of fishing regulations; and e) continuing cooperative research with resource users [22]. While monetary costs associated with implementing outreach and education programs have been assessed [28], outcomes of these efforts, including perceptions of their effectiveness by agencies and the public, have not been fully evaluated.

We developed a novel evaluative framework using mixed methods to assess outreach and engagement efforts related to ESA-listed rockfishes in Puget Sound, from 2010 to 2022. For the purpose of this study, we distinguish *outreach* that is primarily a one-way flow of information from agencies to the public from *engagement* activities that involve more bidirectional communication and collaboration [29]. Our objectives were to: (1) summarize the scope, content, goals, and intended audiences for rockfish-related outreach and engagement; (2) examine practitioner perspectives regarding the effectiveness and challenges of rockfish-related outreach and engagement; (3) assess anglers’ current understanding about rockfish management status and fishing regulations, drawing comparisons to the time of the ESA listings (2010) where possible; and (4) triangulate results from interviews and surveys to synthesize strengths and weaknesses of past outreach and engagement and to provide guidance for future efforts.

## Methods

### Overview of mixed methods approach

We followed a mixed methods framework, using triangulation [30] to bring together a mix of data types and methods to address the study objectives. Triangulation allows for comparison and complementation of knowledge sources to provide a more holistic understanding of patterns or drivers of a particular phenomenon [30,31]. Qualitative and quantitative data were derived from: 1) a review of publicly available outreach and engagement materials; 2) semi-structured interviews with science and management practitioners; and 3) two surveys of fishery participants (Table 1).

**Table 1:** Description of data used in mixed-methods analysis.

Data Type	Description	Year(s)	Source
Inventory	Inventory of publicly available outreach and engagement materials and actions related to ESA-listed rockfish spanning 2010–2024	2010-2024	This study
Survey (historical)	In-person survey administered at boat launches in Puget Sound	2011	[33, 34]
Survey (current)	Online survey administered to recreational fishery license holders in Puget Sound	2022	This study
Interviews	Semi-structured interviews conducted with practitioners (agency staff and partners) who initiated, funded, coordinated, and/or conducted outreach and engagement activities	2023-2024	This study

The table shows data types and descriptions, years data were collected, and studies that generated the data.

We used a “convergent and holistic triangulation” linking process [31] to: a) identify areas of convergence and divergence between surveys and interviews; b) draw broader contextual and causal insights from interviews; and c) validate and extend the empirical findings through published information (Table 2).

**Table 2:** Overview of mixed methods approach.

Objective of Current Study	Data Type	Linking Approach
1. Goals, audience, and approach for outreach	Inventory, Interviews	Convergent
2. Practitioner perspectives on outreach effectiveness and challenges	Interviews	Convergent-Holistic
3. Anglers’ rockfish and regulatory understanding before and after ESA listing	Survey (historical), Survey (current)	Convergent-Holistic
4. Guidance regarding future outreach efforts	Interviews, Survey (current)	Convergent-Holistic

The triangulated data types described in Table 1 are shown for each study objective. ‘Linking Approach’ describes the purpose of triangulation: convergent triangulation seeks common understanding across multiple data types or sources, while convergent-holistic triangulation seeks both areas of agreement as well as an extension or broadening of knowledge as a result of multiple data types or sources.

### Inventory of outreach and engagement activities

A first step was to compile a comprehensive list of rockfish-related outreach and engagement activities implemented since the ESA listing by state and federal agencies and partner organizations. We performed internet searches (Google) using broad sets of keywords (e.g., “rockfish” AND “Puget Sound”) as well as targeted searches on agency/organization websites (Washington Department of Fish and Wildlife [WDFW], NOAA Fisheries, Pacific States Marine Fisheries Commission, Paua Marine Research Group, Seattle Aquarium, Point Defiance Zoo and Aquarium). Additionally, we drew from coauthors’ knowledge and experience and asked interview participants (see below) with institutional knowledge to provide an overview of outreach and engagement efforts that they or their organizations led or participated in. This served to ground truth our independent searches and fill in gaps, especially for physical outreach materials (e.g., signs at boat ramps, handouts, hats, drink holders).

### Semi-structured interviews

We conducted semi-structured interviews with practitioners who had initiated, funded, coordinated, and/or conducted outreach and engagement activities related to rockfish in the Puget Sound region since the ESA-listing (75 FR 22276). Interview participants included individuals from state and federal agencies, non-governmental organizations (e.g., public aquariums), and fishing associations. We identified potential interviewees based on the coauthors’ professional networks and knowledge of outreach and engagement by NOAA Fisheries and WDFW staff. We recruited additional participants through snowball sampling, in which interviewees recommend other individuals knowledgeable on the topic [32]. Interview questions were designed to document outreach and engagement efforts related to ESA-listed rockfish since 2010 and explore themes related to outreach and engagement approaches, efficacy, and public participation in management (S1 File). For a question about outreach goals, we used the evaluative framework of Beierle [9] to predetermine categories describing social goals of public participation and asked interviewees to identify additional goals as needed. The interview protocol was reviewed and approved by the University of Washington Human Subjects Division of the Institutional Review Board (protocol #STUDY00018553).

Interviews were conducted via videoconference (Aug 2023 – Nov 2024) by the lead author and were audio recorded with written and verbal informed consent of interview participants. Audio recordings were transcribed using a secure online transcription service (Otter.ai) and manually reviewed to correct typographical errors. Transcripts were analyzed using the qualitative coding software Atlas.ti by the lead author. We developed a codebook iteratively, as follows. First, we developed a set of codes drawn directly from the interview questions. These included codes related to the role of the interviewee in ESA listing and recovery and subsequent outreach and engagement efforts; partners involved in these efforts; goals, focus, audience, and evaluation of outreach and engagement; and perceived challenges and assessment of future outreach needs and strategies (Table 3). Each transcript was closely read while listening to the audio to refine the initial set of codes and add new codes inductively, based on topics raised by interviewees.

**Table 3:** Coding scheme used for qualitative analysis of interview data.

Code Group	Code Group Description	Codes Included
Role: Listing and Recovery	Primary role(s) of the interviewee in rockfish listing and recovery planning and implementation.	Administrative, General Awareness, Management, Research
Role: Outreach and Engagement	Primary role(s) of the interviewee in rockfish-related outreach and engagement.	Assisting Other Staff, Supervising Other Staff, Coordinating/ Conducting
Outreach: Goals	Interview guide—Part 2, Q2: What were the goals? Ask specifically whether the social goals of public participation from Beierle (1999) were implicitly or explicitly included. Additional goals were added inductively based on interviewee responses.	Educate Public, Engender Conservation Mindset, Foster Trust, Improve Compliance, Incorporate Public Values, Increase Decision Quality, Increase Interest, Increase Knowledge, Make Cost-Effective Decisions, Political Advocacy, Reduce Conflict
Outreach: Audience	Interview guide—Part 2, Q3: Who was/were the intended audience(s)?	Boat-Based Anglers, Decision-Makers, Divers, General Public (Adults), Non-English Speakers, Recreational Anglers, Shore-Based Anglers, Spear Fishers, Youth
Outreach: Types	Forms of outreach and engagement used.	Citizen Science, Email, Informal Conversations, K-12 Education, One-Way, Permanent Signage, Print Materials, Promotional Items, Public Talks, Social Media, Two-Way, Videos, Website
Outreach: Focus	Specific focus (content) of outreach and engagement.	Ecology, Fisheries, Regulations, Release Methods, Species Identification
Outreach: Evaluation	Interviewee discussed what ‘success’ looks like and/or how to measure outreach/ engagement.	Engagement Metrics, Greater Trust, Improved Knowledge, More Engaged Public, This Study
Partnerships	Catch-all category that captures discussions about formal and informal partnerships among agencies or other groups, etc.	Formal Arrangements, Funding, Informal Collaboration
Challenges	Challenges to effective outreach and engagement. Challenges that arose during outreach and engagement activities.	Broad Audience, Conflicting Goals, COVID, Institutional Barriers, Insufficient Funding, Insufficient Staff/Time, Lack of Clear Roles, Not Reaching Some Audiences, Rockfish Lower Priority
Strategies	Strategies for effective outreach/engagement.	Opportunistic Efforts, Partnerships, Use Existing Materials
Future Needs	Future needs or priorities identified for rockfish-related outreach and engagement.	Applied Research, Expertise, Funding, Hopeful Messaging, Partnerships, Reach More Diverse Groups

Definitions for all codes are in S2 File.

### Surveys

As part of a published baseline study, in-person surveys with boat-based anglers were conducted around the Puget Sound region from July to September 2011 [33,34]. The surveys were designed to evaluate anglers’ awareness of recent ESA-listings of three species of rockfish, knowledge of resulting regulatory changes, understanding of fundamental rockfish biology, ability to distinguish among local species of rockfish, and degree of support for conservation efforts aimed at both rockfishes at-large and ESA-listed rockfish in particular [33,34]. A majority of surveyed anglers were unable to correctly identify species of highest conservation priority, which prompted regulatory agencies and educational organizations to enhance existing outreach efforts and initiate novel programs to increase constituent awareness and engender a conservation mindset.

Building on the approach and results of the baseline study, a new survey was administered by the WDFW in 2022 to licensed recreational anglers in Washington state, using an online survey platform (alchemer.com). Survey questions were designed to assess respondents’ knowledge about rockfish identification and life history; knowledge about the fishery, including awareness of regulations; and exposure to rockfish-related outreach and education efforts over the intervening decade since the baseline study. Additionally, respondents were asked about their fishing experience and activities, including preferred target species and fishing areas. Optional questions to elicit basic demographic information were included. The data did not include any personally identifying information. An excerpt of the survey instrument that includes the questions analyzed in this study is in S3 File. Raw survey data and a code script are in S4 and S5 Files, respectively.

The 2022 survey sampling frame consisted of individuals who held Washington recreational saltwater fishing licenses at any time in the 5 years up to and including the survey year (2018–2022). The database was further restricted to only license holders 18 years of age or older (based on self-reported birth years) and only those with email addresses entered. A survey link was sent directly to the resulting 281,430 email addresses and publicized on the WDFW’s social media accounts. Based on a cursory examination of the contact information in the licensing database, some of the email addresses appeared to contain typographical errors and are unlikely to be valid. Because the WDFW’s email distribution platform was unable to track undeliverable messages, the exact number of successfully delivered emails is unknown. The survey was open from July 12 through September 30, 2022. A total of four contacts were made through email and social media, consisting of an initial email distribution and reminders sent at two months, four weeks, and one week before the survey closed.

Data from separate phone-based surveys of angling effort conducted by WDFW demonstrate that most saltwater anglers in Puget Sound target salmon, and to a much lesser extent, halibut, bottomfish, or other species [35,36]. As such, we expected the number of 2022 survey responses to be a small fraction of the total anglers initially contacted. A total of 4145 survey responses were submitted, with a 52.6% completion rate (i.e., every page of the survey was viewed, but not all questions were necessarily answered by every respondent). Of these, 2179 surveys were completed in full. We restricted the analysis to only completed surveys by anglers who reported fishing at least one year in Puget Sound (n = 2020; i.e., omitted anglers who reported that they fish only in freshwater). We developed a flexible R [37] coding framework to subset and analyze diverse forms of data (qualitative, quantitative, open-ended, closed-ended, multiple choice, single choice). Our analysis focused on a subset of questions related to access to and types of information about bottomfish species identification and fishing regulations; and knowledge about rockfish regulations and handling best-practices (S3 File). We summarized the percentages of respondents who selected each response option.

For a subset of questions (n = 3), responses were compared to those from the 2011 baseline survey conducted just after the ESA listings for rockfish were finalized [33,34] (S3 File). While there was considerable overlap in the themes of other questions between the two surveys, this subset addressed the same specific content.

## Results

In this section, we first describe rockfish-related outreach and engagement activities, based on results from the inventory and interviews. Next, we detail findings from interviews (perspectives of outreach and engagement practitioners) and the online survey (public understanding of rockfish biology and fisheries). Last, we bring together results from interviews and the survey to draw conclusions about outreach and engagement effectiveness and identify future growth opportunities.

### Inventory of rockfish-related outreach and engagement activities

**Fig 1:**
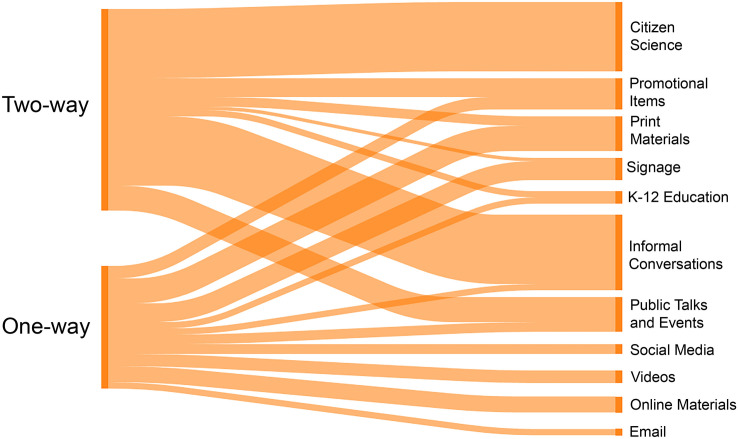
Directionality of information flow associated with outreach and engagement activities. Information flow (left side) is characterized as one-way (outreach) or two-way (engagement) for various types of rockfish-related outreach and engagement activities discussed by interviewees (right side). The width of the bands is proportional to the frequency of coded text segments from interviews for which codes on the left co-occurred with codes on the right.

A wide variety of outreach and engagement efforts were initiated following the ESA listing, focused on providing information about ESA-listed rockfish population status and conservation goals, release strategies, species identification, and ecology (Table 4). These various activities reflected a mix of one-way and two-way communication (Fig 1). Outreach materials (one-way flow of information) included permanent signage at boat launches and marinas, aquarium displays, videos, social media posts, and online and printed materials. The most widely distributed print and online materials were designed to improve rockfish species identification and provide anglers with information on rockfish release strategies that reduce barotrauma (e.g., ~ 10,000 species identification cards and ~20,000 “Protect Washington’s Rockfish” pamphlets distributed to date; Table 4). Most outreach focused on adult recreational anglers and divers, but some efforts aimed to engage a broader audience, including youth (e.g., publication of a children’s book about rockfish and corresponding curriculum guide for teachers [38]). The primary audiences identified by interviewees as a focus of outreach and engagement efforts were: 1) recreational boat-based anglers who target salmon, groundfish, crab, and other marine species; 2) members of recreational fishing organizations (e.g., Puget Sound Anglers); 3) recreational divers who observe and/or harvest marine species underwater; 4) youth (K-12 students); 5) fisheries professionals engaged in Puget Sound research and/or management; and 6) other members of the public who recreate near boat launches or visit local aquariums.

**Table 4:** Outreach and engagement activities related to rockfish fisheries and management in Washington state.

Type	Focus	Audience	Title/Citation	Description	Organization(s) Involved
One-way communication (outreach)					
Print and Online Materials	Species ID; Release Methods	Recreational Anglers	WDFW. 2013. *Protect Washington’s Rockfish*. Online: https://wdfw.wa.gov/sites/default/files/2019-02/protect_rockfish.pdf	Pamphlet on accurately identifying species of concern, reporting catch and release, and effective rockfish release strategies. Over 20,000 distributed to date.	WDFW
Print and Online Materials	Species ID	Recreational Anglers	WDFW. 2019. *Species Identification Card*. Online: https://wdfw.wa.gov/sites/default/files/2019-02/rockfish_species_id.pdf	Identification guide includes WA rockfish and co-occurring species often confused for rockfish. At least 10,000 distributed to date.	WDFW; Pacific States Marine Fisheries Commission; Puget Sound Anglers
Print and Online Materials	Ecology	Youth	Makeyev, C. 2020. *The Rockfish Kids Book: the Secret Lives of Bocaccio and Yelloweye Rockfish*. The Mermaid Scientist. Online: https://mermaidscientist.org/the-rockfish-kids-book	Children’s book about the ecology of ESA-listed rockfish species and corresponding curriculum guide for elementary (K-6) teachers. The book and curriculum align with Next Generation Science Standards. Free to download, and free print copies available to teachers by request.	NOAA Fisheries; The Mermaid Scientist
Print and Online Materials	Species ID	Divers	NOAA Fisheries. n.d. *Young-of-year Rockfishes Citizen Science Survey Guide*. Online: https://media.fisheries.noaa.gov/dam-migration/rockfish_guide_reduced.pdf	Brochure for divers about how to identify ESA-listed juvenile rockfish in Puget Sound. Information on how to collect data and what information to record.	NOAA Fisheries; Seattle Aquarium
Print and Online Materials	Ecology; Fisheries; Species ID; Release Methods	General Public (Adult), Recreational Anglers, Divers	NOAA Fisheries. n.d. *Rockfish Newsletter*. Online. (e.g., https://content.govdelivery.com/accounts/USNOAAFISHERIES/bulletins/3c66260)	Bi-annual newsletter about rockfish research and management.	NOAA Fisheries
Videos	Ecology; Fisheries	General Public (Adults), Youth	Graner, F. 2016. *Recovering ESA-listed Rockfish in Puget Sound* [Video]. Sealife Productions & NOAA Fisheries. Online: https://videos.fisheries.noaa.gov/detail/video/5210169114001/recovering-esa-listed-rockfish-in-puget-sound?autoStart=true&q=rockfish	Video highlighting rockfish ecology and research and recovery measures for rockfish listed under the ESA.	NOAA Fisheries; Sealife Productions
Permanent Signage	Ecology; Species ID; Release Methods	Recreational Anglers	Rockfish information signs; 2017 (created), 2018 (installed)	Permanent, informational signage posted at boat launches throughout Puget Sound on rockfish identification, conservation, and release strategies.	WDFW; NOAA Fisheries; The Seattle Aquarium
Two-way communication (engagement)					
Social Media	Ecology; Fisheries; Species ID; Release Methods	General Public (Adult), Recreational Anglers	Rockfish-related content; 2010-present	Engagement with anglers and community members through periodic posts on social media channels	NOAA Fisheries; WDFW
Public Talks and Events	Ecology; Fisheries	General Public (Adult), Youth, Recreational Anglers, Divers, Fisheries Professionals	Educational presentations on rockfish; 2010-present	Dozens of presentations to angler and diver organizations, school groups, and fisheries professionals.	NOAA Fisheries; Paua Marine Research Group; WDFW
Promotional Items, Informal Conver-sations	Ecology; Fisheries; Species ID; Release Methods	Recreational Anglers	Deepwater descender giveaways and information on species identification; 2010-present	Outreach to anglers to better identify rockfish and use rapid-submergence techniques to reduce the effects of barotrauma. Directly responsive to Recovery Plan goal to “continue or expand the WDFW and Puget Sound Anglers project in which descending devices are purchased and distributed for free or at reduced cost.”	NOAA Fisheries; Puget Sound Anglers; WDFW
Community/ Citizen Science	Ecology	Recreational Anglers, Divers	Collaborative research and community science on Puget Sound rockfish (various projects^1^^,^^2^^,^^3^^,^^4^^,^^5^)	Partnerships among agencies, NGOs, recreational fishers, and SCUBA divers to collect data and measure recovery of ESA-listed rockfish species. Research on rockfish life history, movement behavior, recruitment, and population structure.	NOAA Fisheries; Northwest Straits Initiative; Paua Marine Research Group; Point Defiance Zoo and Aquarium; Puget Sound Anglers; REEF; Seattle Aquarium; The Sea Doc Society; WDFW

Activities are categorized according to their type, specific focus, and intended audience. ESA = Endangered Species Act, NOAA = National Oceanic and Atmospheric Administration, WDFW = Washington Department of Fish and Wildlife

^1^ Andrews, K.S., K.M. Nichols, A. Elz, N. Tolimieri, C.J. Harvey, R. Pacunski, D. Lowry, K.L. Yamanaka, D.M. Tonnes. 2018. Cooperative research sheds light on population structure and listing status of threatened and endangered rockfish species. Conservation Genetics 19: 865−878. https://doi.org/10.1007/s10592-018-1060-0

^2^ Andrews, K., K. Nichols, C. Harvey, N. Tolimieri, A. Obaza, R. Garner, and D. Tonnes. 2019. All hands on deck: Local ecological knowledge and expert volunteers contribute to the first delisting of a marine fish species under the Endangered Species Act. Citizen Science: Theory and Practice 4(1), https://doi.org/10.5334/cstp.221

^3^ NOAA Fisheries. n.d. Cooperative and Citizen Science on Puget Sound Rockfish. Online: https://www.fisheries.noaa.gov/cooperative-and-citizen-science-puget-sound-rockfish

^4^ NOAA Fisheries. n.d. Science Leading to Recovery and Delisting of Puget Sound Rockfish. Online: https://www.fisheries.noaa.gov/west-coast/science-data/science-leading-recovery-and-delisting-puget-sound-rockfish

^5^ Obaza, A., D. Lowry, J. Selleck, K.S. Andrews, and A.O. Shelton. 2023. Young-of-the-year rockfish monitoring plan for the Southern Salish Sea. In National Marine Fisheries Service, West Coast Region, Seattle, WA. 29 + App. Online: https://www.fisheries.noaa.gov/s3/2023-05/yoy-rockfish-monitoring-plan-2023.pdf

Engagement approaches (two-way flow of information) offered opportunities for dialogue among managers, scientists, fishers, and divers (Table 4). These included dozens of presentations by agency staff to fishing and diving organizations and at community events, targeted distribution of deepwater descenders to anglers, and funding and technical support for community (or citizen) science initiatives. A young-of-the-year rockfish monitoring plan, which included a survey protocol for use by community scientists and local organizations, was developed collaboratively by non-governmental organizations and agency partners [39,40]. A notable collaborative research effort by NOAA Fisheries, recreational anglers, and divers generated new genetic information that led to the delisting of canary rockfish (82 FR 7711, [26,27]).

### Perspectives from outreach and engagement practitioners (interviews)

#### Summary of interview participants.

We interviewed eight key informants for this study who led regional efforts in outreach and engagement related to ESA-listed rockfishes. These interview participants represented the core group of state and federal agencies, environmental NGOs, and fishing organizations that coordinated and facilitated rockfish-related outreach and engagement activities. One interviewee identified their gender as female, and seven as male. All identified as white and ranged in age from mid-40s to mid-60s.

#### Goals and actions.

**Fig 2:**
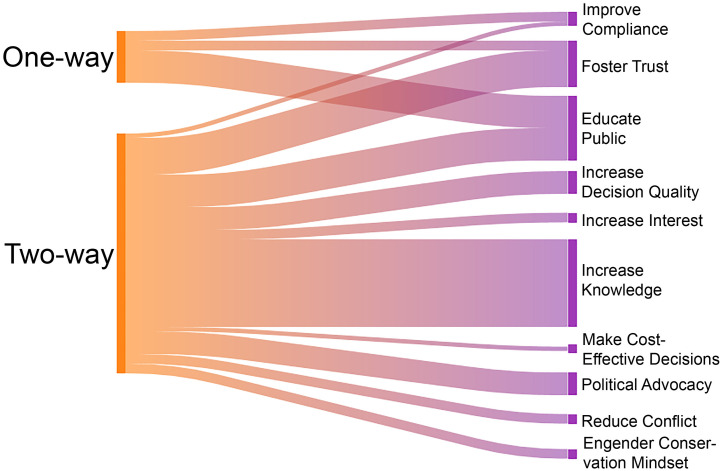
Directionality of information flow associated with outreach and engagement goals. Information flow (left side) is characterized as one-way (outreach) or two-way (engagement) for rockfish-related activities that addressed a range of goals discussed by interviewees (right side). The width of the bands is proportional to the frequency of coded text segments from interviews for which codes on the left co-occurred with codes on the right.

Rockfish-focused outreach and engagement efforts were typically designed to address multiple goals, including all social goals of public participation described by Beierle [9], as well as a few additional goals identified by interviewees (Table 5). The most commonly identified outreach and engagement goals (by 6 or more participants) were to educate the public, increase the substantive quality of organizational or management decisions, foster trust in institutions, improve compliance with regulations, and reduce conflict (Table 5). However, some goals emerged as more prominent than others based on the extent to which interview participants discussed them. These more salient goals included increasing collective awareness and knowledge about rockfish, educating the public, and fostering trust (Fig 2). These goals were addressed through a variety of methods, including outreach activities that primarily relied on one-way flow of information and engagement that emphasized bidirectional communication among practitioners and their intended audience (Fig 2, Table 4).

**Table 5:** Social goals of public participation identified by interview participants as goals for rockfish-related outreach and engagement.

Social Goal	Definition	N	Quote
Educate Public	Educating the public was a social goal of the outreach and engagement efforts.	8	“…they [fishers] would bring up a rockfish that’s like, 50 centimeters long, you know—I mean, *big* fish—and you tell them that only 50% of those individuals that are that big are reproductively active. And you could just see light bulbs going off…like you could just see their eyes go wide, and say ‘Wow, I used to fill up those big garbage cans full of rockfish, like every day back in the ‘80s’. And when they hear things like that, they’re like, oh, it’s gonna take a long time to recover.” (Agency Staff, Interview #4)
Increase Decision Quality	Increasing the substantive quality of decisions was a social goal of the outreach and engagement efforts.	7	“The more you know, the better decision making you do. If you don’t have that input, you kind of do a disservice to your constituency if you’re just basing your decisions on what you know without any other context. I am really tied into that because I am a consumptive user, so I want to make sure that my concerns and needs are being met on that side.” (Agency Staff, Interview #2) “We had so many tremendous data gaps. I mean, we just we didn’t know how many fish were out there. We didn’t know exactly where they were, we didn’t know what the bycatch rates were. … And I felt like if we had pursued MPAs out of the gate, it would be through such ignorance and not having relationships with key stakeholders, and the Tribes as well. So, [a project involving engagement with anglers] was really designed to fill those data gaps about what people know about rockfish and how they behave as fisher people.” (Agency Staff, Interview #8)
Foster Trust	Fostering trust in institutions was a social goal of the outreach and engagement efforts.	6	“And so very early on, I think, I started realizing, I can’t be *such* a scientist that I’m afraid to talk to people, I’m afraid to meet people where they need to be met on these different ideas, or else they’re not going to support spending money on species recovery or they’re not going to support ecosystem restoration.” (NGO Scientist, Interview #5)
Improve Compliance*	Improving compliance with or buy-in regarding fisheries and wildlife regulations was a social goal of the outreach and engagement efforts. Added as category in interview guide.	6	“A lot of that was, as you might expect, it was outreach. It wasn’t engagement, per se. It was, here’s the new rule published in the pamphlet, published online, published in flyers, handouts, anywhere that we could get it out to people so that they would just see it, and know that there had been a closure. Yeah, so phase one was just to stop everybody taking rockfish” (Agency Staff, Interview #1)
Reduce Conflict	Reducing conflict (e.g., among stakeholders, between government and stakeholders) was a social goal of the outreach and engagement efforts.	6	“Once people have an understanding of the challenges you face, I think it makes them more comfortable to have a conversation, and they’re not so confrontational. And yeah, again, it’s just being honest and being transparent. I mean, reducing that confrontation level is key to getting things accomplished.” (Agency Staff, Interview #2)
Incorporate Public Values	Incorporating public values, assumptions, and preferences into decision making was a social goal of the outreach and engagement efforts.	5	“I think when you leave the community out of decisions, they aren’t always embraced and followed. So getting the public’s perception about management is important.” (NGO Scientist, Interview #6) “…we’re largely on the same team, we all want the same thing. We want healthy rockfish populations and the people that we’re working with on this program are just trying to document those changes to affect better management and policy.” (NGO Scientist, Interview #7)
Make Cost-Effective Decisions	Making decisions cost-effectively was a social goal of the outreach and engagement efforts.	5	“Absolutely. Because we were always working with limited resources and we knew it.” (Agency Staff, Interview #1) “…cooperative research was also a way for us to help develop better relationships and think about what are our funding priorities in the immediate future.” (Agency Staff, Interview #8)
Increase Knowledge*	Engagement and collaboration to generate new knowledge and/or share information about rockfish biology/ecology and fisheries. Category added inductively.	5	“We’ve had folks [community science volunteers] that have come and say, I don’t really feel confident collecting data. I don’t know what I’m looking at. And it’s like, ‘Okay, I don’t care, come dive! Let’s look at rockfish together’... and then you form a relationship, and you start looking at rockfish, and then they build a little bit of confidence, and then…they develop enough confidence to start collecting data.” (NGO Scientist, Interview #7)
Engender Conservation Mindset*	Shift towards more conservation-oriented goals and attitudes as a result of outreach/engagement. Increase public interest in rockfish for non-consumptive use and inspire appreciation for or curiosity about rockfish. Category added inductively.	4	“We were giving talks at aquariums and fishing clubs and dive associations and anybody that would let us come talk about rockfish, because we were then kind of shifting gears a bit to say, look, we’ve stopped your ability to interact with the species from a fishery standpoint, you can’t bring it home fillet it, put it on a plate anymore. But gosh, these are still pretty amazing species…So we were really trying to talk to people about non-exploitative uses of the fish, you know, how can you still interact with them? How can you still know what they are, learn to identify them, all these different things, and appreciate them without bringing them home to you?” (Agency Staff, Interview #2)
Political Advocacy*	Goal of increasing awareness and institutional commitment (funding, staff, etc.) among lawmakers, politicians, and agency leads. Outreach to agency staff to influence policy outcomes. Category added inductively.	3	“Well, we jumped on it, because we said, ‘Well, if you’re not at the table, you’re on the table,’ you know. And so we…told [the agency] we were going to bring our descender program into the Puget Sound. And we did and we started buying descenders and handing them out everywhere and explaining to people. Then NOAA wanted to make it a law that you had to have a descender and use it. I said, okay, now that we’ve got everybody with descenders, we want to open water back up [for fishing].” (Fisher, Interview #3)

The categories marked with an asterisk were added to the interview guide prior to interviews or inductively through the coding process and are not among the social goals described by Beierle [9]. N is the total number of participants who reported each goal and exemplary quotes are provided for illustration.

#### Challenges and strategies.

Based on recollections of interview participants—and evident from the outreach and engagement inventory (Table 4)—only a small group of individuals and organizations led a majority of the outreach and engagement efforts. Outreach and engagement activities were guided by the broad strategies outlined in the Rockfish Recovery Plan (see Introduction) and supported through consistent, low-level funding for outreach to support recovery efforts. While NOAA Fisheries provided funding and staff support to these activities, no full-time positions at either state or federal agencies were dedicated to rockfish-related outreach and engagement. This was identified as the primary challenge by a majority of interviewees, particularly state and federal agency staff who described feeling spread thin by the combined responsibilities of scientific research and monitoring, promulgating new regulations, and education and outreach to support the Rockfish Recovery Plan. Many interviewees described their rockfish-focused outreach efforts as opportunistic and done in conjunction with other work, such as giving talks while traveling for fieldwork or meetings and staffing agency booths at fairs, fishing and hunting expos, and trade shows. One agency staff person painted a vivid picture of the effort involved:

“We were averaging 14 talks a year and I would say that was throughout the greater Puget Sound area…So yeah, we were doing a lot of driving. We were coordinating that largely with field work, though. So you know, it was kind of wild…there were times where I would leave early on a Monday to go do [field work] for four days, I would get off late on a Thursday and on the way south, I would stop somewhere on a Friday and give a lecture, give another lecture on Saturday morning, give another one on Saturday night, and then be home Sunday morning.” (Interview #1)

In addition to limited staff time and funding, another key challenge discussed by interviewees was the complexity of reaching a large, diverse audience throughout the region. Most outreach efforts were directed towards boat-based recreational anglers (Table 4), and some interview participants recognized the need to broaden their reach to shore-based anglers, fishers for whom English is not a primary language, and other groups. This was described by an agency staff member:

“I think we should be making more efforts to reach out to more of these diverse communities that we’re now seeing starting to participate in fisheries…So, you know, a lot of these folks that we see fishing, they may have not been exposed to any of this [ESA rockfish issues], so how do we reach them?” (Interview #2)

Some interview participants also noted the challenges of communicating science and conservation issues to the public when there are more pertinent or engaging issues at the forefront. A rindsearcher made a light-hearted comparison to media coverage of sports to highlight the challenges of science communication:

“We get an article in the paper, and we’re like, oh, this is great, people are gonna be educated. But look at the newspaper during March Madness—there are 20 stories on basketball. And people are talking about it all the time. People are not talking about rockfish! So we’ve got to at least get something in the conversation where people start talking about that.” (Interview #5)

Even within a fisheries context, rockfish and other bottomfish are generally a lower priority to the recreational angling community compared to salmon [19,35,36], and this was reflected by an interview participant who noted that “a lot of the anglers that I talked to at the ramp aren’t really bottomfish fishermen. Mostly, they’re salmon fishermen” (Interview #2). Continuously encouraging interest and care for rockfish among fishers and the broader public was seen as a persistent challenge when rockfish fisheries have been closed for a decade in much of Puget Sound.

Some interview participants who work for NGOs talked about the sometimes difficult balance between science and advocacy in their work and maintaining credibility in light of broader concerns around mistrust in science. One researcher described the challenge, saying,

“I feel like there’s such distrust in so many institutions right now. I definitely want [our organization] to be a trusted voice. … We try to only bring our voice to, say, management or policy issues, if it’s supported by data and if it meets our mission, and then once we commit to it, even if we get backlash, we stay committed. But we’re very careful about how we use our voice in different circumstances so that we do remain a trusted institution” (Interview #6).

Interviewees noted that collaborative science provided an important avenue for building and maintaining trust (Table 5).

All interviewees discussed the importance of partnerships for meeting outreach goals. Most partnerships were informal collaborations, rather than formal arrangements as part of the rockfish recovery effort. In many instances, partnerships were solidified through funding for specific projects, largely from NOAA Fisheries to other entities (Table 4). Collaboration was highly valued by interview participants. An agency staff person described the importance of collaborative research with fishers and divers to advance knowledge about rockfish, saying,

“Working with [anglers] and some of the dive groups to develop these information streams, they’ve jumped on board to help us. And, you know, if we hadn’t had a lot of that help, we wouldn’t have gotten there. You know, canaries would probably still be on the list.” (Interview #2)

A researcher involved in a collaborative project with fishers talked about the importance of conversations on the boat while fishing for rockfish (almost 80 sampling days over two years [27]) as a catalyst for building trust and shared understanding:

“So it’s like those individual moments where, like you’re listening to their [fishers’] stories and understanding how important fishing is to them. And then you can reciprocate by giving a little bit of context to why this species is gonna have a hard time recovering. Like, that’s my punchline, I guess for this whole thing—being on the water, understanding why fishing is important, and engaging with them in a place that’s comfortable—then you see the fish and you can start to understand.” (Interview #4)

The projects that interviewees highlighted relied on leadership and coordination among fishing and diving organizations, government agencies (state, federal, and tribal), public aquariums, and other environmental NGOs.

Some of the early collaborative, community-based research done while the Rockfish Recovery Plan was under development served to identify partnership opportunities and help prioritize future outreach and engagement efforts. One agency staff person described the role of baseline study [33], saying,

“…several questions that [the researcher] asked anglers were designed to get their preferences for rockfish recovery priorities. And that was a good way for us to find the low hanging fruit, where we agree. The science agrees, and the key stakeholder group agrees, that we should pursue descending devices, avoiding derelict fishing gear, things like that. We’re just like, we all agree on that, let’s partner on that, and we can work out the more difficult stuff down the line.” (Interview #8).

In this example, community engagement through collaborative research was a strategy for tuning and prioritizing ongoing outreach efforts.

### Public understanding of rockfish biology and fisheries (surveys)

#### Summary of survey respondents.

Surveys were completed by 2,020 anglers who reported having fished between 1 and 80 years in Puget Sound, with an average of 24.5 years (SD 18.5) of fishing experience per respondent. Some respondents reported membership in a fishing association (15.1%) or environmental organization (16.5%). Modes of fishing included motorized boats (85.0%), shore-based (31.6%), piers (18.9%), and non-motorized boats (14.9%). Just 2.9% of respondents identified as a guide or charter operator. Most respondents typically target or catch salmon (83.9%) and crab (77.7%), with fewer targeting or catching lingcod (51.4%), halibut (26.1%), rockfish (16.2%), and other bottomfish (34.2%). Respondents were asked to select marine areas (MA 5–13) in which they typically fish. Admiralty Inlet (MA 9) was most frequently (40.3%), and South Puget Sound (MA 13) least frequently (16.6%), identified by respondents as their typical fishing area.

Demographic data were provided for 75.8% of completed surveys by Puget Sound anglers (n = 1532). Survey respondents identified predominantly as male (90.9%) and white (83.9%), with a fairly even distribution of ages between 35 and 75 years (84% fell within this range). Respondents were primarily residents of King County (22.9%), Snohomish County (14.2%), and Pierce County (11.1%), corresponding largely with human population centers with access to saltwater fishing in the region [41].

#### Awareness of ESA-listed rockfishes.

Survey respondents were asked to select the species of rockfish that are listed under the ESA in the Puget Sound region from a multiple choice list that included the three species that were originally listed in 2010 as endangered (bocaccio) or threatened (yelloweye rockfish; canary rockfish, since delisted), along with five common rockfish species that are not ESA-listed (black, brown, copper, quillback, and yellowtail rockfishes). Respondents were also given the option of ‘none.’

A majority of the survey respondents who answered this question (n = 1981) selected yelloweye rockfish (79.8%, n = 1580) and canary rockfish (65.5%, n = 1297) as ESA-listed, while only 37.0% (n = 733) selected bocaccio. The other rockfishes were selected by 25.6–38.9% (n = 508–771) of respondents, depending on the species (S3 File). Just 6.3% (n = 125) of respondents selected ‘none.’ In comparison, the 2011 survey showed that of 536 respondents who answered an open-ended question about which species of rockfish are listed under the ESA, 32.6% (n = 175) named yelloweye rockfish, 18.1% (n = 97) named canary rockfish, and 3.2% (n = 17) named bocaccio [33]. A majority of respondents in 2011 (65.3%, n = 350) did not know that any rockfish were ESA-listed [33].

#### Knowledge of regulations.

A series of survey questions were designed to evaluate anglers’ knowledge about rockfish regulations. Respondents were asked to identify the timing of bottomfish recreational fishing seasons in Puget Sound from a list of options. Of those who responded to the question (n = 1509), just over a quarter (25.6%, n = 387) correctly indicated that bottomfishing is open year-round, with restricted seasons for lingcod and a handful of other species. Close to half of respondents (48.7%, n = 735) selected options that described open seasons in spring and summer, with restrictions for particular species. A smaller percentage (7.4%, n = 111) selected options that described open season in spring and summer, with no species-specific restrictions. The remaining respondents indicated they did not know the timing of bottomfish seasons (18.3%, n = 276).

Another survey question asked respondents to select all of the bottomfishing regulations currently in effect in Puget Sound (Marine Areas 5–13). Nine options were provided, including six correct regulations and 3 incorrect regulations, as well as an option for ‘unknown’. Awareness of fishing regulations varied among specific regulation types. Of the 1,996 respondents who answered the question, just over half (54.8%, n = 1094) were aware that no retention of rockfish (any species) is allowed in Puget Sound marine areas. This is slightly lower than the percentage of 2011 survey participants who were aware that rockfish retention was not permitted (63.7%, n = 341; [33]).

#### Knowledge of rockfish handling best-practices.

Rockfish are susceptible to barotrauma when brought to the surface quickly, so concerted outreach was aimed at educating anglers about the benefits and proper use of deepwater descending devices (hereafter, ‘descenders’). Descenders come in a variety of forms designed to return a fish to depth quickly and recompress affected organs and tissues with the goal of reducing barotrauma injuries. While descenders are the required method of releasing rockfish when targeting bottomfish and halibut (WAC 220-310-110 (5)), other release methods, such as dehooking and rapidly returning to the water, can also be effective at minimizing mortality depending on the species and fishing depth. Survey questions were aimed at evaluating anglers’ current handling practices and views on descenders.

In response to a question about rockfish release methods, respondents (n = 1993) could select multiple answers from a list of options (S3 File). A small majority of respondents reported that they use descenders (57.4%, n = 1144), close to half dehook in the water (46.1%, n = 919), and over a quarter dehook on deck (28.8%, n = 573) before returning the fish to the water. Overall, 86.6% of respondents (n = 1726) reported using one of the three most effective release methods (i.e., use of descenders and/or rapid dehooking and release). In comparison, 2011 survey responses to the same question (n = 534) indicated low use of descenders (4.3%, n = 23), with a majority of respondents dehooking in the water (67.5%, n = 361) and some dehooking on deck (12.7%, n = 68).

In regulation (WAC 220-310-120) and as described in the WDFW regulations pamphlet (https://www.eregulations.com/assets/docs/resources/WA/24WAFW_LR8.pdf), anglers are instructed not to puncture the swim bladder of a rockfish using a sharp object (also referred to as “fizzing” or “venting”) before releasing. However, a small percentage of survey respondents (5.5%, n = 110) reported that they do puncture the fish before release. This was similar to the percentage of 2011 survey respondents reporting that they puncture the swim bladder (5.2%, 28 of 534 total responses; [33]).

Respondents were asked to indicate one or more types of descenders they carry while bottomfishing (total of 1988 responses). An inverted, barbless hook (e.g., Shelton fish descender) was the most common descending device used by respondents (42.1%, n = 837), followed by an automatic pressure release (e.g., Seaqualizer) (24.9%, n = 495). Some respondents indicated that they do not carry descenders (12.1%, n = 240) and a small percentage reported using other methods, such as an inverted crate or tug release (<5% each).

Respondents were asked to select a statement that best described their views on descenders from a list of options (n = 1471 responses). A majority of respondents (74.2%, n = 1092) believe that descending devices are effective for getting rockfish to the bottom; 28.9% (n = 425) used descending devices before they were required, and 45.3% (n = 667) started using them when they were mandated. The survey itself seemed to help raise awareness of descenders for some people; 20.9% (n = 308) selected the option “I hadn’t heard about descending devices before this survey.” Only 4.8% (n = 71) of respondents do not think descenders work to get rockfish to the bottom.

#### Access to information about rockfish and regulations.

To help guide future outreach efforts, the survey examined whether and how information about rockfish identification and fishing regulations provided by state and federal agencies was reaching fishers. The survey included questions about the sources and types of information fishers were aware of and found to be most effective.

More than half of the respondents (56.2%, n = 1122) reported using the WDFW website for fish identification information. Use of other online and print materials for fish identification, and consulting with friends and family, were also common [29.3–36.4% of respondents (n = 585–726) per source]. A majority of respondents consult the WDFW website (79.1%, n = 1584), or more specifically the online WDFW regulations booklet (65.9%, n = 1320), for current fishing regulations. Many also reference the paper regulations booklet (59.5%, n = 1191). Reported use of the Fish WA app (39.6%, n = 793) and consulting news releases for regulation changes (32.0%, n = 640) were less common.

Prior to completing the survey, a majority of respondents had seen rockfish posters and signs at boat launches (74.4%, n = 1309) and species identification cards (64.8%, n = 1139). Other outreach materials, such as brochures, keychain species guides, drink holders, and presentations, were less commonly recognized [1.9–20.4% (n = 34–359) of respondents]. Respondents indicated that the most effective places to distribute rockfish identification materials are boat ramps (74.0%, n = 1474) and local bait or dive shops (61.9%, n = 1233). A variety of other outreach approaches, including distribution of materials at fishing piers and dive sites, email, and online sources were also viewed as effective by one-third or more of respondents.

### Synthesis of outreach and engagement successes and challenges

Taken together, the interviews and surveys indicate strengths and challenges of past rockfish-related outreach and engagement efforts, and highlight strategies for the future. Here, we triangulate the data sources (Table 1) to summarize key take-aways from over a decade of outreach and engagement efforts.

**Fig 3:**
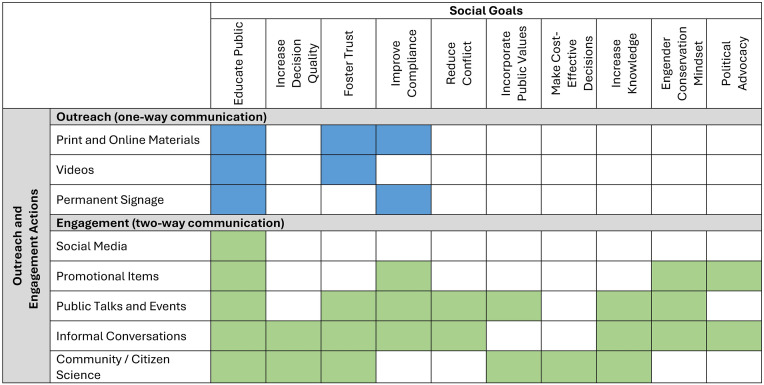
Portfolio of rockfish-related outreach and engagement activities and the social goals of public participation in ESA-listed rockfish recovery that they addressed. Outreach activities are in blue shaded cells and engagement activities are in green shaded cells, as identified from practitioner interviews and angler surveys. Social goals are arranged in order from most (left) to least (right) frequently identified goals of rockfish-related outreach and engagement by interview participants. Print and online materials include species identification guides and pamphlets; newsletters; and K-12 educational materials.

A strength of the overall outreach and engagement approach used by agency staff and collaborators was maintaining clear goals and focus, while using a wide variety of tools to reach a broad audience. Since the time of the rockfish listings in Puget Sound, specific outreach and engagement activities have played different roles in addressing social goals of public participation in rockfish conservation (Fig 3). The survey responses indicated the importance of signage at boat launches and online and print materials for conveying information on rockfish biology, species identification, and fishing regulations. These types of one-way approaches to information sharing were identified by interview participants as reaching a larger audience but meeting far fewer social goals than two-way approaches (Fig 2).

Investing in both education and knowledge co-production can reach a broad audience while building trust with key stakeholders. In this case, the substantial effort put towards education of anglers (largely one-way communication) about rockfish biology and fisheries showed some measure of success based on survey results and interviews. The survey results showed that across multiple metrics, anglers’ awareness of the listed rockfish species, as well as knowledge and use of deepwater descenders, had increased over the decade following the ESA listings.

Interviewees spoke about the value of in-person engagement, such as public presentations and discussions, conversations with anglers at the dock, and generating shared knowledge through collaborative science and the “lightbulb moments” that can arise from these projects. One agency staff person noted a particular aspect of rockfish biology that sparked the most interest from the public, saying,

“One of the most amazing things is when you tell people how old rockfish get, they’re like, ‘What?!’ They have no clue. And so really, I think that is the switch that gets people to start thinking in a different light. You know, they go, ‘Oh, I did not know that.’ And so that’s really a great springboard into that conversation.” (Interview #2)

While community science and collaborative research often involves a relatively small number of individuals, the high quality of engagement can have a ripple effect by activating networks of knowledge sharing within the larger community. Additionally, this type of engagement was important for informing rockfish recovery and monitoring efforts.

All of the interview participants identified the challenges of limited staff capacity and funding in meeting outreach and engagement goals. Yet, all spoke positively about the necessary partnerships and collaborations that were created as a result. Future efforts would benefit from institutional investment in dedicated outreach and engagement staff, as well as information technology and communications support to ensure availability of up-to-date information on websites, social media, and newsletters. Funding and staff support can fluctuate with administration changes and institutional priorities, so collaboration and opportunistic efforts will continue to be important moving forward. The outreach strategy outlined in the Rockfish Recovery Plan aims to ensure some degree of continuity, despite such variability [22].

## Discussion

Our analysis supports three main findings with respect to future rockfish-related public engagement in Puget Sound: 1) a portfolio of outreach and engagement approaches will likely be most effective for reaching a diverse audience and meeting multiple social goals; 2) community-engaged collaborations allow for a deeper discourse that builds trust, knowledge, and shared stewardship; and 3) strategic partnerships and capacity-building will continue to be important for meeting outreach and engagement goals. These results can help guide future efforts that are tailored to best meet the evolving needs of rockfish recovery. From this study, and our experience, many anglers and divers in Puget Sound have a vested interest in the long-term conservation of rockfish and are willing to follow fishing regulations, contribute to research, and participate actively in management efforts that aspire to that same goal.

### Out of sight, out of mind: conservation challenges for depleted species

Substantial challenges in meeting social goals of public participation, and building the knowledge needed to support rockfish management and conservation, include historical declines in rockfish abundance and reduced access to rockfish arising from current fishery closures. Multiple interviewees noted the difficulty of raising awareness and interest in rockfish when people are no longer able to fish for them and are encouraged to avoid encounters with rockfishes through both regulation and education. This is a common challenge for conservation of endangered, threatened, and otherwise depleted species. Biodiversity loss and declines in species abundance have led to erosion of local ecological knowledge, including identification of plants and animals [42] and knowledge of their uses, especially for youth [43,44]. Even at the time of the ESA-listing for Puget Sound rockfish species, population declines over decades had led to shifting baselines in perception of rockfish abundance, where younger generations recollected relatively high abundances of rockfish during periods in which older generations had already observed depletion [45].

Resource users’ understanding of the links between a species’ ecology and management measures aimed at protecting habitats or key life history stages has been associated with greater buy-in of conservation measures (e.g., [46]), justifying continued educational efforts aimed at increasing the public’s ecological knowledge. A study of recreational anglers in Puget Sound found that prior fishing experience for rockfish was associated with knowledge of rockfish life history and a higher degree of support for rockfish recovery measures [34]. Thus, addressing one outreach and engagement goal, such as educating the public, can have positive indirect effects on other goals, such as increasing compliance. Continued efforts to raise awareness about rockfish biology, ecology, and populations are valuable for educating anglers and a broader public, but they cannot replace recreational fishers’ experiential and relational knowledge that commonly inspires stewardship action [47]. It is also important to note that not all interactions with rockfish in Puget Sound are associated with harvest, and substantial efforts to engage divers in research, underwater photography, and other non-consumptive activities continue to enhance public awareness and interest [10,39].

A portfolio of outreach and engagement approaches may be necessary for raising public awareness of, interest in, and support for conservation of rockfishes and other “rare and little-known species” [48] that lack salience due to their scarcity, lack of social or economic importance, or other factors. Using a mix of one-way and two-way communication strategies targeted to a set of key audiences (e.g., [29]) may also increase the likelihood of meeting a broader suite of outreach and education goals [49]. While casting a wide net may be warranted in resource management contexts in which target audiences are broad and diverse and there is limited public awareness of the management issue, it can be difficult to sustain without dedicated staff and funding. More targeted approaches to specific audiences, such as community-based social marketing, may be beneficial for motivating behavioral change [50] or increasing the success of fundraising campaigns for less salient species [51].

### Engendering a conservation mindset: a shift from compliance to stewardship

Natural resource management agencies commonly conduct outreach to the public with the goals of raising awareness, educating constituents, encouraging compliance with rules, and supporting future participation in management processes (e.g., [52,53]. Increasing the public’s understanding of, and compliance with, regulations is especially important when there is limited capacity for enforcement, which is common for recreational fisheries [54–56]. While initial outreach goals for ESA-listed rockfish were largely focused on compliance with fishing regulations, over time these efforts moved towards fostering interest in rockfish and conservation action. Angler associations in the Puget Sound region played an important role in promoting use of deepwater descenders and engaging fishers in broader stewardship efforts, stemming from both a conservation ethic and the desire to rebuild rockfish populations to reopen fisheries. Distribution of deepwater descenders at no charge to anglers likely played a role in promoting their use, as popular models may be cost-prohibitive to some (e.g., $60 USD for a SeaQualizer). A fishery participant interviewed in this study described these efforts as a proactive demonstration of shared stewardship, which could both influence policy and garner greater support for recreational fisheries. Similarly, in Alaska rockfish fisheries, voluntary use of deepwater descenders by charter fishing associations led to a regulatory change that required their use on recreational vessels [57]. Conservation actions that address ecological goals and increase political capital for fishers are a component of environmental stewardship that is aimed at ensuring access to fisheries for future generations [58].

Outreach and engagement that promotes dialogue and relationship-building among fishers and fishery practitioners is likely to serve a wider range of social goals than one-way information flow [59]. However, strategically placed signage at areas frequented by fishers and divers, informational pamphlets, and online resources can be effective at reaching much larger audiences, as indicated by our survey results. In British Columbia, Canada, a similar suite of outreach and education approaches were found to increase angler compliance with Rockfish Conservation Areas [56]. Community events that include live demonstrations of best handling practices and species identification could enhance current outreach efforts (e.g., [60]). Mandatory angler education programs associated with licensing have been implemented in some parts of Europe, but are uncommon elsewhere [60].

### Community-engaged research to meet social and ecological goals

A large body of scholarship documents the numerous benefits of knowledge co-production in resource management settings, including greater relevance and applicability of scientific research [61,62], relationship-building and trust [63], and improved conservation outcomes [64]. Various forms of collaborative, cooperative, and community-based participatory research can contribute to broader social-ecological learning among diverse resource users, scientists, and managers [61,65,66]. However, the success of these research partnerships can be diminished if goals and expectations are poorly aligned among participants [67]. Co-production may be strengthened through a process of equitable exchange, in which attention is given to issues of representation, recognition, compensation, data ownership, and reciprocity among partners [68,69].

In this study, interview participants highlighted successes of collaborative science among fishers and agency, NGO, and university researchers for addressing ecological and social goals. Of particular note, the genetic data generated through a collaborative project that involved NOAA scientists, nine recreational fishing guides, and over 100 volunteer anglers led to the first delisting of a marine species (canary rockfish) under the ESA (82 FR 7711; [26,27]). Interviewees described the value this project had in building trust and rapport among scientists and fishers. Ongoing volunteer diver surveys documenting rockfish young-of-the-year [10,39] were also highlighted as an important focus of continued research investment.

### Broadening the scope of participation and engagement for rockfish recovery

Fishery management in the Puget Sound region is jurisdictionally layered, with state, federal, and tribal entities working in coordination as co-managers. During development of the federal Rockfish Recovery Plan, the Northwest Indian Fisheries Commission designated three tribal representatives to serve on the Rockfish Recovery Team [22]. Of importance to Tribes was the assurance that treaty rights to fish in Usual and Accustomed Areas would not be impinged by marine spatial management, such as designation of marine protected areas for rockfish conservation [70,71]. The implementation of outreach and community engagement as part of the Rockfish Recovery Plan was largely carried out by federal and state agency staff. Several interview participants, all non-Indigenous researchers, expressed a desire to develop stronger partnerships with Tribes and Indigenous communities in future rockfish conservation and collaborative research. These research partnerships would be best supported through active engagement of individuals who act as boundary spanners between Indigenous communities and scientific organizations [69].

### Mixed methods as a tool for evaluating outreach and engagement

The mixed methods evaluative framework used here offers a unique approach for integrating quantitative data from surveys with resource users with qualitative data from interviews with practitioners. While the methods and resulting data each had their own limitations, when brought together they provided a more holistic picture of the effectiveness of rockfish-related outreach and engagement. Limitations of the qualitative evaluation process include the time-intensive nature of interviews and associated thematic analysis. This process was feasible given the small group of practitioners who conducted most of the outreach and engagement, but it may not be in another management context with many more participants. The comparison of previous (2011) and recent (2022) surveys is imperfect, given differences in the sampling frames and how surveys were administered before and after outreach interventions; however, the repeated survey questions allowed for some valuable comparisons of angler perspectives before and after the ESA-listings. Attributes of the 2022 online survey could be improved, including reducing the number and complexity of questions, which may have discouraged survey completion in some cases (47.4% of surveys were unfinished). While there are known challenges with online survey methods, a benefit is that they can reach a large number of prospective respondents and yield large sample sizes [72,73]. For future applications of the survey, providing it in multiple languages, disseminating it by mail and email, and tracking the response rate could increase the robustness of results and broaden participation by underrepresented demographic groups. The convergent and holistic linking approach clarified areas of agreement among data sources, which increased validity and reliability of results, while also revealing gaps in particular perspectives on public participation in rockfish recovery.

Many agency efforts to engage the public occur during a formal decision-making process. In the United States, for example, environmental decision-making at the federal level is guided by the National Environmental Policy Act and other laws [e.g., Endangered Species Act (ESA)] that require a formal public process, with opportunities for the public to provide input on management alternatives [74]. After initial agency decisions are made, public engagement can be more ad hoc and not dictated by law or a formal process, but is often intentional, adaptive, and specifically outlined in decision implementation. With funding limitations and shifting priorities, agencies may be forced to choose the forms and focus of outreach based largely, or entirely, on cost and staff capacity. The evaluative framework we demonstrated here could be applied in contexts beyond rockfish conservation to identify the outreach and engagement actions that are most effective for meeting a range of social goals and to guide future prioritization of outreach efforts by agencies.

## Conclusions

Public participation in conservation and management of rockfishes will continue to be important for reaching recovery goals. In the most recent five-year status review for bocaccio and yelloweye rockfish, a priority action identified was to “evaluate efforts to increase angler awareness of fisheries regulations, knowledge of rockfish life history, and species identification ability by conducting systematic surveys of the angling public” and refine the outreach strategy accordingly ([28], p. 39). This study is a first step towards responding to that call and provides a foundation for evaluating the outcomes of future outreach and engagement efforts. Even with limited staff capacity, some additional metrics of participation could be recorded to demonstrate the reach of outreach and engagement, such as the number of public presentations or events, numbers of attendees, and analytics for tracking online engagement. A simple evaluation survey could be developed to elicit participant feedback after public presentations and outreach events. In addition, a simplified version of the online angler survey could be repeated over time. Finally, the qualitative framework we developed for linking outreach and engagement goals and approach with outcomes could be modified for use with focus groups composed of resource users, researchers, management practitioners, and outreach coordinators to improve the inclusion of diverse perspectives and knowledge in rockfish recovery efforts.

## Supporting information

S1 File Semi-structured interview guide. (DOCX)

S2 File Codebook used for qualitative analysis of interview data. (DOCX)

S3 File Subset of survey questions analyzed in this study and data summaries. Summary tables and figures show the distribution of responses.
(DOCX)

S4 File Angler survey data. Metadata (first tab) and de-identified raw survey data (second tab) from an online survey of anglers are included for the subset of survey questions analyzed in this study.
(XLSX)

S5 File R programming code file. Code used to summarize angler survey data.
(R)
