# Supplementary material for: Social-ecological goals and outcomes of public engagement for recovery of endangered and threatened rockfishes (Sebastes spp.)
Source: PLoS One. 2025 Sep 9;20(9):e0331686. doi: 10.1371/journal.pone.0331686 (PMC12419659; doi:10.1371/journal.pone.0331686)
Supplement: S1 File — (DOCX) [file pone.0331686.s001.docx]

**S1 File. Semi-structured interview guide.**

*This interview protocol is a guide for the interviewer, who will conduct verbal, in-person or remote interviews with participants in a semi-structured format. Participants will not have to provide written answers to questions.*

*Reminders for interviewer: Summarize the consent form for the participant and allow time to look it over (includes project overview, confidentiality information, and how long the interview should take). Allow time for questions. Ask them for oral consent. Ask for consent to audio record. If yes, start audio recorder and state date and interview #.*

Part 1. Background and relevant experience

1. What type of organization do you currently work for? For how long?

- Federal agency
- State agency
- Tribe
- Non-profit / NGO
- Academic institution
- Other (specify)

Number of years: ____________

1. What type of organization did you work for at the time of the rockfish ESA-listing (2009-2010)? For how long?

- Federal agency
- State agency
- Tribe
- Non-profit / NGO
- Academic institution
- Other (specify)

Number of years: ____________

1. How would you describe your role in the rockfish ESA listing, recovery planning, and implementation process?
   1. Was this part of your formal job duties?

Part 2. Rockfish outreach and engagement

1. What type(s) of outreach and engagement did you or your organization do related to rockfish in Puget Sound? [Alternate wording: How would you describe your contribution or efforts related to rockfish outreach and engagement in Puget Sound?]
   1. For how long / what years?
   2. How was this effort funded?
2. What were the goals?
   1. Ask specifically whether the following goals were *implicitly* or *explicitly* included (from Beierle 1999 - social goals of public participation):

- educating the public (educate active public and/or inform wider public?)
- incorporating public values, assumptions, and preferences into decision making
- increasing the substantive quality of decisions
- fostering trust in institutions
- reducing conflict
- making decisions cost-effectively
- improving compliance with regulations [AB added]
  1. Do you think your organization was successful in meeting these goals? Why or why not?

1. Who was/were the intended audience(s)? Do you think your organization was successful in reaching this audience? Why or why not?
   1. Are there particular audiences or stakeholder groups that you think should have been a stronger focus of engagement?
2. How would you assess the overall effectiveness of the outreach efforts?
   1. What went well?
   2. What were the challenges?
3. Was there any formal assessment or evaluation of outreach effectiveness?
   1. If yes: what types of evaluative metrics have you used to assess effectiveness?
4. What do you think is needed in terms of future outreach and engagement related to rockfish?
   1. If multiple ideas are provided, ask interviewee how they would prioritize them
5. How do you think future outreach and engagement should be supported (e.g., agency funding/staff, external partners/grant funding, volunteer efforts, etc.)?

Part 3. Demographic information

*Interviewee may write responses to this section if they wish.*

1. In what city or town do you live?
2. In what year were you born?
3. What is your gender?
4. What is your race, ethnicity, or cultural background?
