# Supplementary material for: Social-ecological goals and outcomes of public engagement for recovery of endangered and threatened rockfishes (Sebastes spp.)
Source: PLoS One. 2025 Sep 9;20(9):e0331686. doi: 10.1371/journal.pone.0331686 (PMC12419659; doi:10.1371/journal.pone.0331686)
Supplement: S2 File — (DOCX) [file pone.0331686.s002.docx]

**S2 File. Codebook used for qualitative analysis of interview data.**

| **Code Group** | **Code Group Description** | **Code** | **Code Definition** |
| --- | --- | --- | --- |
| Role: Listing and Recovery | Primary role(s) of the interviewee in rockfish listing and recovery planning and implementation. | Administrative | Administrative duties including providing funding and supervising staff, implementing policies, etc. |
|  |  | General Awareness | Generally aware of listing and related activities, but not directly involved. |
|  |  | Management | Management duties including participation on recovery plan team, creating agency policies, and/or setting regulations. Also includes policy development and political advocacy. |
|  |  | Research | Research duties including conducting fieldwork, collecting data, managing or curating data, provisioning data, and/or analysis. |
| Role: Outreach and Engagement | Primary role(s) of the interviewee in rockfish-related outreach and engagement. | Assisting Other Staff | Assisting with outreach led by other staff in their organization or partner organization. |
|  |  | Coordinating / Conducting | Taking a lead role in coordinating and conducting outreach. |
|  |  | Supervising Other Staff | Supervising other staff who conduct outreach in their organization or partner organization. |
| Outreach: Goals | Interview guide—Part 2, Q2: What were the goals? Ask specifically whether the social goals of public participation from Beierle (1999) were implicitly or explicitly included. Additional goals were added inductively based on interviewee responses. | Educate Public | Educating the public (active public and/or wider public) was a social goal of the outreach and engagement efforts. One of Bierle's (1999) social goals of public participation. |
|  |  | Engender Conservation Mindset | Shift towards more conservation-oriented goals and attitudes as a result of outreach/engagement. Category added inductively. |
|  |  | Foster Trust | Fostering trust in institutions was a social goal of the outreach and engagement efforts. One of Bierle's (1999) social goals of public participation. |
|  |  | Improve Compliance | Improving compliance with or buy-in regarding regulations was a social goal of the outreach and engagement efforts. Category added inductively. |
|  |  | Incorporate Public Values | Incorporating public values, assumptions, and preferences into decision making was a social goal of the outreach and engagement efforts. One of Bierle's (1999) social goals of public participation. |
|  |  | Increase Decision Quality | Increasing the substantive quality of decisions was a social goal of the outreach and engagement efforts. One of Bierle's (1999) social goals of public participation. |
|  |  | Increase Interest | Increase public interest in rockfish for non-consumptive use, e.g., diving, viewing at aquariums, learning natural history, etc. Inspire appreciation for or curiosity about rockfish. Category added inductively. |
|  |  | Increase Knowledge | Engagement and collaboration to generate new knowledge and/or share information about rockfish biology/ecology and fisheries. Category added inductively. |
|  |  | Make Cost-Effective Decisions | Making decisions cost-effectively was a social goal of the outreach and engagement efforts. One of Bierle's (1999) social goals of public participation. |
|  |  | Political Advocacy | Goal of increasing awareness and institutional commitment (funding, staff, etc.) among law makers, politicians, and agency leads. Outreach to agency staff to influence policy outcomes. Category added inductively. |
|  |  | Reduce Conflict | Reducing conflict (e.g., among stakeholders, between government and stakeholders) was a social goal of the outreach and engagement efforts. One of Bierle's (1999) social goals of public participation. |
| Outreach: Audience | Interview guide—Part 2, Q3: Who was/were the intended audience(s)? | Boat-Based Anglers | Intended audience was boat-based anglers. |
|  |  | Decision-Makers | Intended audience was law makers, politicians, and agency leads. |
|  |  | Divers | Intended audience was scuba divers. |
|  |  | General Public (Adults) | Intended audience was broad public, largely focused on adults. |
|  |  | Non-English Speakers | Intended audience was non-English speakers. |
|  |  | Recreational Anglers | Intended audience was recreational anglers, unspecified. |
|  |  | Shore-Based Anglers | Intended audience was shore-based anglers. |
|  |  | Spear Fishers | Intended audience was spear fishers. |
|  |  | Youth | Intended audience was youth. |
| Outreach: Types | Forms of outreach and engagement used. | Citizen Science | Collaborative science with community members and/or stakeholders. |
|  |  | Email | Information sent by email to stakeholders (intermittent or regular). |
|  |  | Informal Conversations | Conversations with public in informal settings (e.g., at boat launches, informally at public meetings, on social media, etc.). |
|  |  | K-12 Education | Engagement with K-12 teachers and students via curriculum development, providing print materials (e.g., rockfish kids' book), classroom visits, etc. |
|  |  | One-Way | Unidirectional flow of information (e.g., from agency to stakeholder). |
|  |  | Permanent Signage | Signs created and posted in public spaces, e.g., marinas and boat launches. |
|  |  | Print Materials | Print materials such as flyers, trifold pamphlets, newsletters, etc. |
|  |  | Promotional Items | Give-aways, such as cup holder with rockfish information, rockfish ID keychains, deepwater descenders, etc. |
|  |  | Public Talks | Public presentations about rockfish ecology, management, conservation, etc. |
|  |  | Social Media | Posts created on social media channels, such as Facebook, Twitter, etc. |
|  |  | Two-Way | Bidirectional flow of information (e.g., dialogue between agency staff and stakeholders). |
|  |  | Videos | Videos created to provide information about rockfish ecology, management, conservation, etc. |
|  |  | Website | Website provides information about rockfish ecology, management, conservation, etc. |
| Outreach: Focus | Specific focus (content) of outreach and engagement. | Ecology | Rockfish biology, life history, habitat, etc. |
|  |  | Fisheries | Rockfish fisheries, management, history of conservation issues. |
|  |  | Regulations | Communicating regulations to anglers, such as through the regs books, online, etc. |
|  |  | Release Methods | Teaching people about deepwater descender requirements, how to use them, science about their effectiveness, etc. |
|  |  | Species ID | Species identification, focusing on rockfish. |
| Outreach: Evaluation | Interviewee discussed what 'success' looks like and/or how to measure outreach / engagement. | Engagement Metrics | Quantitative metrics of engagement, such as number of attendees at talks, number of public events held, social media engagement, etc. |
|  |  | Greater Trust | Demonstration of outreach effectiveness is improved trust in the management agency and staff. |
|  |  | Improved Knowledge | Demonstration of outreach effectiveness is improved knowledge among public audiences about rockfish ecology, biology, conservation, regulations, etc. |
|  |  | More Engaged Public | Demonstration of outreach effectiveness is more engaged public in discussions about and interest in rockfish ecology, biology, conservation, regulations, etc. Demonstration of curiosity, care, and stewardship. |
|  |  | This Study | Interviewee said that this study is an evaluation of outreach/engagement effectiveness. |
| Partnerships | Catch-all category that captures discussions about formal and informal partnerships among agencies or other groups, etc. | Formal Arrangements | Descriptions of formal roles and responsibilities of various agencies in rockfish research, management, enforcement, and outreach efforts. |
|  |  | Funding | Funding was moved from one organization to another for outreach. Dedicated funding provided for outreach and engagement. |
|  |  | Informal Collaboration | Descriptions of how agencies and other organizations are collaborating on rockfish research, management, and outreach efforts. Primarily informal arrangements, not required by law. |
| Challenges | Challenges to effective outreach and engagement. Challenges that arose during outreach and engagement activities. | Broad Audience | Reaching out to a large, broad audience posed a challenge to outreach and engagement. Identifying the target audience was a challenge. |
|  |  | Conflicting Goals | Agency mandates and outreach goals sometimes contradictory, e.g., trying to reduce rockfish encounters while increasing people's knowledge of rockfish. |
|  |  | COVID | Challenges created by the COVID pandemic and subsequent changes in society (e.g., more reliance on remote participation, less time, etc.). |
|  |  | Institutional Barriers | Barriers to meeting outreach goals due to restrictive policies or guidelines, administrative hurdles, bureaucratic inertia, or institutional culture. |
|  |  | Insufficient Funding | Lack of dedicated funding or not enough funding to address outreach/education goals. |
|  |  | Insufficient Staff/Time | Staff and/or staff time not dedicated to outreach specifically. Often a driver of opportunistic efforts. |
|  |  | Lack of Clear Roles | Unclear what entity is responsible for carrying out outreach. |
|  |  | Not Reaching Some Audiences | Challenge meeting outreach goals because some intended audiences or groups were left out, hard to reach or insufficient effort expended to reach them, or structural inequalities. |
|  |  | Rockfish Lower Priority | Challenge meeting outreach goals because rockfish are not of interest or a priority to either agency, stakeholders, or both. |
| Strategies | Strategies for effective outreach/engagement. | Opportunistic Efforts | Conducted outreach opportunistically in conjunction with other job duties, like fieldwork, or building on other parallel efforts. |
|  |  | Partnerships | Developed partnerships with other organizations to meet or broaden outreach goals. Leveraged funding, ongoing collaborations, and existing professional networks. |
|  |  | Use Existing Materials | Used existing outreach materials created by other organizations, such as videos, websites, or educational materials. |
| Future Needs | Future needs or priorities identified for rockfish-related outreach and engagement. | Applied Research | Research and its practical application to address both outreach and conservation goals. |
|  |  | Expertise | Expertise and dedicated staff needed to meet future goals. |
|  |  | Funding | Funding needed to meet future goals. |
|  |  | Hopeful Messaging | More hopeful, forward-looking messages will help improve outreach and conservation outcomes. Need for storytelling and creative communication approaches. |
|  |  | Partnerships | Partnerships needed to meet future goals. |
|  |  | Reach More Diverse Groups | More effort needed to increase reach of outreach/engagement, esp. to diverse audiences or underrepresented groups. |
