# Supplementary material for: Social-ecological goals and outcomes of public engagement for recovery of endangered and threatened rockfishes (Sebastes spp.)
Source: PLoS One. 2025 Sep 9;20(9):e0331686. doi: 10.1371/journal.pone.0331686 (PMC12419659; doi:10.1371/journal.pone.0331686)
Supplement: S3 File — Summary tables and figures show the distribution of responses. (DOCX) [file pone.0331686.s003.docx]

**S3 File. Subset of survey questions analyzed in this study and data summaries.** Summary tables and figures show the distribution of responses.

Questions and associated responses are shown for both the 2022 survey (this study) and 2011 survey (Sawchuk 2012) where applicable.

Citation: Sawchuk JH. Angling for insight: examining the recreational fishing community’s

knowledge, perceptions, practices, and preferences to inform rockfish recovery planning in Puget Sound, Washington. Master of Marine Affairs thesis. University of Washington, Seattle, Washington, USA; 2012.

**2022 Survey; Question 2**. Which species of rockfish are listed under the Endangered Species Act for the Puget Sound region? Select all that apply.

Response options:

a. Black Rockfish

b. Bocaccio (**correct**)

c. Brown Rockfish

d. Canary Rockfish

e. Copper Rockfish

f. Quillback Rockfish

g. Yelloweye Rockfish (**correct**)

h. Yellowtail Rockfish

i. None

Total respondents: 1981

| **Response**  **Selected** | **Number of**  **Respondents** | **Proportion of**  **Respondents** |
| --- | --- | --- |
| Yelloweye Rockfish | 1580 | 0.798 |
| Canary Rockfish | 1297 | 0.655 |
| Copper Rockfish | 771 | 0.389 |
| Bocaccio | 733 | 0.370 |
| Quillback Rockfish | 693 | 0.350 |
| Yellowtail Rockfish | 568 | 0.287 |
| Black Rockfish | 549 | 0.277 |
| Brown Rockfish | 508 | 0.256 |
| None | 125 | 0.063 |

**2011 Survey; Question 22.** Do you know which species of rockfish are listed on the Endangered Species list in Puget Sound/San Juan Islands? If yes, will you please list them?

Total respondents: 536

| **Response**  **Provided** | **Number of**  **Respondents** | **Proportion of**  **Respondents** |
| --- | --- | --- |
| Yes, respondent stated they know ESA-listed species | 172 | 0.321 |
| Yes, could name Yelloweye Rockfish | 175 | 0.327 |
| Yes, could name Canary Rockfish | 97 | 0.181 |
| Yes, could name Bocaccio | 17 | 0.032 |
| Yes, respondent knew all ESA-listed rockfish | 17 | 0.032 |
| Stated yes but could not name or incorrectly named species | 155 | 0.289 |
| No, respondent stated they did not know listed species | 350 | 0.653 |

**2022 Survey; Question 5**. To the best of your knowledge, what best describes the general annual timing of bottomfish recreational fishing seasons in Puget Sound?

Response options:

a. Open only May through August

b. Open April through September, with restricted seasons for Lingcod and a few other species

c. Open year-round, with restricted seasons for Lingcod and a few other species (**correct**)

d. Closed year-round for most species, but open June through September for Lingcod and Pacific Halibut

e. Closed October through February, but open for all species March through September

f. I don’t know

Total respondents: 1509

| **Response Selected** | **Number of**  **Respondents** | **Proportion of**  **Respondents** |
| --- | --- | --- |
| a. Open only May through August | 92 | 0.061 |
| b. Open April through September, with restricted season… | 238 | 0.158 |
| c. Open year-round, with restricted seasons for Lingcod… | 387 | 0.256 |
| d. Closed year-round for most species, but open June through… | 497 | 0.329 |
| e. Closed October through February, but open for all species… | 19 | 0.0126 |
| f. I don’t know | 276 | 0.183 |

**2022 Survey; Question 6**. To the best of your knowledge, which of the bottomfishing regulation(s) listed below are currently in effect in Puget Sound (Marine Areas 5-13 on the map)? Select all that apply.

Response options:

a. No restrictions on bottomfish species, gear, or depth

b. Keep 1 rockfish per day

c. Keep 1-3 rockfish per day, but only Blue/Deacon and Black Rockfish, and only in Area 5 (**correct**)

d. No retention of Yelloweye Rockfish or Bocaccio (**correct**)

e. No retention of rockfish of any species (**correct**)

f. No fishing deeper than 120 ft. while salmon or halibut fishing

g. No fishing deeper than 120 ft. while bottomfish fishing (**correct**)

h. A descending device must be onboard (**correct**)

i. A descending device must be onboard and rigged for use (**correct**)

j. I don’t know

Total respondents: 1996

| **Response Selected** | **Number of**  **Respondents** | **Proportion of**  **Respondents** |
| --- | --- | --- |
| e. No Retention | 1094 | 0.548 |
| h. Descender Onboard | 854 | 0.428 |
| g. Depth Lim Bottomfish | 846 | 0.424 |
| i. Descender Rigged | 774 | 0.388 |
| d. No Yelloweye Bocaccio | 747 | 0.374 |
| c. One To Three Lim Spp Area | 356 | 0.178 |
| j. Unknown | 318 | 0.159 |
| f. Depth Lim Salmon Halibut | 148 | 0.074 |
| b. One Per Day | 110 | 0.055 |
| a. No Restrictions | 18 | 0.009 |

**2011 Survey; Question 21.** What are the current fishing regulations in Puget Sound/San Juan Islands? Check all that apply.

Response options:

a. Keep 1 rockfish per day

b. No fishing deeper than 120 ft. while salmon or halibut fishing

c. No fishing deeper than 120 ft. while bottomfishing

d. No retention of rockfish

e. Do not know

Total respondents: 535

| **Response**  **Selected** | **Number of**  **Respondents** | **Proportion of**  **Respondents** |
| --- | --- | --- |
| Keep 1 rockfish per day | 30 | 0.056 |
| No fishing deeper than 120 ft. while salmon or halibut fishing | 5 | 0.093 |
| No fishing deeper than 120 ft. while bottomfishing | 120 | 0.224 |
| No retention of rockfish | 341 | 0.637 |
| Do not know | 156 | 0.292 |

**2022 Survey; Question 8.** Which method(s), if any, have you used when releasing rockfish? Select all that apply.

Response options:

a. I have never caught a rockfish

b. Cut the line as soon as the fish is recognized/identified

c. Dehook and release without removing the fish from the water

d. Remove the fish from the water to dehook, then release

e. Puncture swim bladder (fizzing/venting) before releasing

f. Sink fish quickly using a descending device designed to release it at depth (including homemade devices)

g. Other - Write In (Required)

Total respondents: 1993

| **Response**  **Selected** | **Number of**  **Respondents** | **Proportion of**  **Respondents** |
| --- | --- | --- |
| f. Descender | 1144 | 0.574 |
| c. Dehook Water | 919 | 0.461 |
| d. Dehook Deck | 573 | 0.288 |
| a. Never Caught | 290 | 0.146 |
| e. Puncture | 110 | 0.055 |
| g. Other | 94 | 0.047 |
| b. Cut Line | 22 | 0.011 |

**2011 Survey; Question 18.** Which method(s) do you use when releasing accidentally caught rockfish? Check all that apply.

Response options:

a. Dehook and release without removing the fish from the water

b. Puncture swim bladder (fizzing)

c. I have never caught a rockfish

d. Sink fish quickly using a device designed to release it at depth

e. Remove the fish from the water to dehook, then release

Total respondents: 534

| **Response**  **Selected** | **Number of**  **Respondents** | **Proportion of**  **Respondents** |
| --- | --- | --- |
| Dehook and release without removing the fish from the water | 361 | 0.675 |
| Puncture swim bladder (fizzing) | 28 | 0.052 |
| I have never caught a rockfish | 72 | 0.135 |
| Sink fish quickly using a device designed to release it at depth | 23 | 0.043 |
| Remove the fish from the water to dehook, then release | 68 | 0.127 |
| Other | 90 | 0.168 |

**2022 Survey; Question 9.** What type of descending device (a device used to sink a fish and release it at depth) do you carry when you go fishing for bottomfish or halibut in Puget Sound? Select all that apply.

Response options:

a. I don’t fish for bottomfish or halibut

b. I hadn’t heard about descending devices before this survey

c. I don’t carry a descending device

d. Automatic pressure release (e.g., Seaqualizer)

e. Inverted, barbless hook (e.g., Shelton Fish Descender, self-rigged)

f. Tug-released or bottom contact jaw clamp (e.g., RokLees, modified Fish Grip, Blacktip)

g. Weighted, upside-down milk crate on a line

h. Other - Write In (Required)

Total respondents: 1988

| **Response**  **Selected** | **Number of**  **Respondents** | **Proportion of**  **Respondents** |
| --- | --- | --- |
| e. Inverted Hook | 837 | 0.421 |
| d. Auto Pressure Rel | 495 | 0.249 |
| a. No Bottomfish | 458 | 0.230 |
| c. Do Not Carry | 240 | 0.121 |
| b. Unaware | 195 | 0.098 |
| f. Tug Release | 98 | 0.049 |
| g. Crate | 58 | 0.029 |
| h. Other | 55 | 0.028 |

**2022 Survey; Question 10.** Which statement best describes your thoughts about descending devices?

Response options:

a. I hadn’t heard about descending devices before this survey

b. I think they work to get rockfish to the bottom, and I used/carried them before they were required when bottomfish/halibut fishing.

c. I think they work to get rockfish to the bottom, but I only started using/carrying them because they are required when bottomfish/halibut fishing.

d. I don’t think they work to get rockfish to the bottom, and I only use/carry them because they are required when bottomfish/halibut fishing.

e. I don’t think they work to get rockfish to the bottom, so I don’t use/carry them.

Total respondents: 1471

| **Response**  **Selected** | **Number of**  **Respondents** | **Proportion of**  **Respondents** |
| --- | --- | --- |
| a. I hadn’t heard about descending devices before this … | 308 | 0.209 |
| b. I think they work to get rockfish to the bottom, and I used… | 425 | 0.289 |
| c. I think they work to get rockfish to the bottom, but I only… | 667 | 0.453 |
| d. I don’t think they work to get rockfish to the bottom, and I only use… | 46 | 0.0313 |
| e. I don’t think they work to get rockfish to the bottom, so I don’t… | 25 | 0.017 |

**2022 Survey; Question 26.** What resource(s) do you typically consult to identify fish, either while fishing or at home? Select all that apply.

Response options:

a. I rely on my fishing buddies/friends/family for species identification

b. I rely on my memory

c. WDFW websited. General internet search (Google, Bing, etc.)

e. A fish identification app (please specify which)

f. Species identification posters/signs at boat launches

g. Quick reference sheet or dive card (i.e., single card with basic fish pictures and names)

h. Keychain species guide

i. Fish identification book (e.g., field guidebook with descriptions, not just pictures)

j. Other(specific websites or books welcomed)

Total respondents: 1997

| **Response**  **Selected** | **Number of**  **Respondents** | **Proportion of**  **Respondents** |
| --- | --- | --- |
| c. WDFW Website | 1122 | 0.562 |
| g. Reference Sheet | 726 | 0.364 |
| i. Fish ID Book | 718 | 0.360 |
| d. Internet Search | 675 | 0.338 |
| b. Memory | 626 | 0.313 |
| a. Friends | 585 | 0.293 |
| f. Boat Launch Posters | 496 | 0.248 |
| j. Other | 164 | 0.082 |
| h. Keychain Guide | 65 | 0.033 |
| e. App | 57 | 0.029 |

**2022 Survey; Question 27.** What resource(s) do you consult to check current fishing regulations? Select all that apply.

Response options:

a. WDFW website

b. WDFW Sportfishing Rules Pamphlet (online)

c. WDFW Sportfishing Rules Pamphlet (paper copy)

d. Fish Washington App

e. WDFW news releases

f. Sportfishing websites (e.g., Puget Sound Anglers, NW Sportsman Magazine)

g. I rely on my memory from previous years’ regulations

Total respondents: 2002

| **Response**  **Selected** | **Number of**  **Respondents** | **Proportion of**  **Respondents** |
| --- | --- | --- |
| a. WDFW Website | 1584 | 0.791 |
| b. WDFW Regs Online | 1320 | 0.659 |
| c. WDFW Regs Paper | 1191 | 0.595 |
| d. Fish WA App | 793 | 0.396 |
| e. News Release | 640 | 0.320 |
| f. Sportfish Websites | 165 | 0.082 |
| g. Memory | 97 | 0.048 |

**2022 Survey; Question 28.** Which of the following rockfish outreach materials have you seen before this survey? Select all that apply.

Response options:

a. Keychain species guide

b. Rockfish posters/signs at boat launches

c. L’il Sucker suction drink holder

d. Species identification card

e. Rockfish trifold brochure

f. Oral presentations given by WDFW/NOAA staff at meetings or in public venues

g. Other - Write In (Required)

Total respondents: 1759

| **Response**  **Selected** | **Number of**  **Respondents** | **Proportion of**  **Respondents** |
| --- | --- | --- |
| b. Posters | 1309 | 0.744 |
| d. Species ID Card | 1139 | 0.648 |
| e. Brochure | 359 | 0.204 |
| g. Other | 174 | 0.099 |
| a. Keychain Guide | 115 | 0.065 |
| f. Presentation | 62 | 0.035 |
| c. Drink Holder | 34 | 0.019 |

**2022 Survey; Question 29.** In order to reach anglers/spearfishers such as yourself, where do you think is the most effective place to post and/or distribute rockfish identification materials like those in the previous question? Select all that apply.

Response options:

a. Local bait/dive shops

b. Boat ramps

c. WDFW offices

d. WDFW/NOAA/other partner website

e. Fishing piers/Dive sites

f. Boat/Trade shows

g. Physical mail

h. E-mail

i. Digital newsletters

j. Social media

k. Other - Write In (Required)

Total respondents: 1993

| **Response**  **Selected** | **Number of**  **Respondents** | **Proportion of**  **Respondents** |
| --- | --- | --- |
| b. Boat Ramps | 1474 | 0.740 |
| a. Bait & Dive Shops | 1233 | 0.619 |
| e. Piers | 1101 | 0.552 |
| h. Email | 965 | 0.484 |
| d. Agency Websites | 709 | 0.356 |
| j. Social Media | 639 | 0.321 |
| g. Mail | 605 | 0.304 |
| f. Trade Shows | 589 | 0.296 |
| i. Newsletters | 575 | 0.289 |
| c. WDFW Offices | 309 | 0.155 |
| k. Other | 200 | 0.100 |
